# Low-nutrient diet in *Drosophila* larvae stage causes enhancement in dopamine modulation in adult brain due epigenetic imprinting

**DOI:** 10.1098/rsob.230049

**Published:** 2023-05-10

**Authors:** J. M. Zúñiga-Hernández, Gonzalo H. Olivares, Patricio Olguín, Alvaro Glavic

**Affiliations:** ^1^ Laboratorio Biología del Desarrollo, Departamento de Biología, Facultad de Ciencias, Universidad de Chile, Chile; ^2^ Programa de Genética Humana, ICBM, Biomedical Neuroscience Institute, Departamento de Neurociencia, Facultad de Medicina, Universidad de Chile, Chile; ^3^ Escuela de Kinesiología, Facultad de Medicina, Center of Integrative Biology (CIB), Universidad Mayor, Chile

**Keywords:** nutrients, diet, *Drosophila*, epigenetics, behaviour, development

## Abstract

Nutrient scarcity is a frequent adverse condition that organisms face during their development. This condition may lead to long-lasting effects on the metabolism and behaviour of adults due to developmental epigenetic modifications. Here, we show that reducing nutrient availability during larval development affects adult spontaneous activity and sleep behaviour, together with changes in gene expression and epigenetic marks in the mushroom bodies (MBs). We found that open chromatin regions map to 100 of 241 transcriptionally upregulated genes in the adult MBs, these new opening zones are preferentially located in regulatory zones such as promoter-TSS and introns. Importantly, opened chromatin at the Dopamine 1-like receptor 2 regulatory zones correlate with increased expression. In consequence, adult administration of a dopamine antagonist reverses increased spontaneous activity and diminished sleep time observed in response to early-life nutrient restriction. In comparison, reducing *dop1R2* expression in MBs also ameliorates these effects, albeit to a lesser degree. These results lead to the conclusion that increased dopamine signalling in the MBs of flies reared in a poor nutritional environment underlies the behavioural changes observed due to this condition during development.

## Graphical abstract

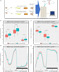

## 1. Introduction

Within the high range of challenging conditions that a developing organism may face, nutrient scarcity is a frequent adverse scenario [1]. Even though several mechanisms operate to manage this condition during organ growth, the adult state could inherit long-lasting changes in metabolism, and how the cells use their energy, due to epigenetic modifications in stem cells during developmental stages [1–3]. One of the organs with more versatile and significant adaptations to the lack of nutrients is the central nervous system (CNS), which is also very sensitive to environmental changes during development, making metabolic [4], cellular [5] and physiological adaptations [6] to overcome the nutrient shortage. In humans, fetal undernutrition causes long-lasting changes in CNS, thus adults tend to develop cognitive disorders such as schizophrenia or bipolar disorders, even though after receiving complete or normal nutrition during childhood and adulthood [7,8]. Similarly, in rodents, undernutrition *in utero* leads to alterations in adult behaviour, inflammatory profile in the liver, and metabolic diseases in adulthood, such as insulin resistance and obesity [1]. In *Drosophila melanogaster*, neuroblasts from fasting larvae overexpress *Alk*, a tyrosine kinase receptor that keeps the insulin/PI3K pathway active, while the other organs turn it off [4].

The consequences of early-life malnutrition at the cellular and behavioural level depend on the stage where the lack of nutrients occurs. In *Drosophila,* the brain develops from one hundred neuroblasts that appear at the first round of neurogenesis at embryonic stage 9. Neuroblasts divide until the onset of the larval stage generating in each division a ganglion mother cell (GMC) and a neuroblast. Neuroblasts divide again, generating two neurons each, giving rise to neurons and forming the primary lineage [9]. The second lineage originates after a brief pause of the primary neuroblast expansion and takes place from larval to late pupal stages. During this second expansion, the organism does not depend on yolk resources, but the nutrients ingested from the environment [9]. Thus, the nutritional conditions during the second expansion stage could modify neuroblasts epigenetic configuration and lineage, thus affecting the synapsis generated and their excitability, leading to behavioural disorders [5,10–13]. However, studying this issue in specific types of neurons could be challenging. Although rodent models allow the study of gene function, RNA expression and epigenetics, the techniques commonly used are frequently unsuitable for analysing specific neurons and directly relate their changes to behavioural alterations. By contrast, *Drosophila melanogaster* is suitable for testing epigenetic and transcriptional changes even in neuronal subpopulations in response to early-life nutritional scarcity and its correlation with behavioural changes [14]. Here, we study the transcriptional and epigenetic changes in a specific neuronal population of the Mushroom bodies (MBs) in response to early-life nutrition restriction and their potential consequences for adult behaviour. MBs is a conserved brain structure among insects composed of and adult neuropile [15] that consist of around 2000 Kenyon cells (KCs) per lobule. KCs divide into three main neuronal types (*α*/*β*, *α*′/*β*, *γ*) [16]. MBs development begins with the second neuroblast expansion phase in the larval stage, in which *γ* KCs differentiate before third instar larvae, and the *α*/*β* and *α*′/*β* KCs differentiate during the late third instar and pupae stages [17]. The MBs are involved in associative learning and complex behaviours such as walking [18], feeding [19], olfactory memory [20,21] and sleep [22,23]. MBs receive dopaminergic innervation from different neuropils such as PAM dopamine neurons that project from the superior medial protocerebrum (SMP) and crepine neuropil (CRE) [24]. KCs harbour the highest expression of dopamine receptors [25,26], which are G protein-coupled receptors. Their activation increases neuron excitability by promoting calcium currents and inhibition of potassium channels [25]. The role of the MBs in locomotor activity and sleep behaviour relies on dopamine signalling [27]; thus, an imbalance in dopamine signalling may lead to altered behaviours.

In this work, we show that nutritional restriction in the larval period promotes motor activity and reduces the sleep time of adult flies. Moreover, mRNA-seq and ATAC-seq analyses of KCs revealed increased expression and open chromatin configurations in genes coding for elements related with assembly of pre-synaptic vesicles, vesicle release, cation transport and dopamine signalling. Finally, we found that pharmacological or genetical reductions of dopamine signalling in adults diminished the behavioural alterations induced, indicating that dopamine is a major player in long-term behavioural alterations derived from malnutrition during the developmental phase of the brain.

## 2. Results

### 2.1. Developmental nutrient restriction increases adult locomotion and reduces sleep time

**Figure 1.**
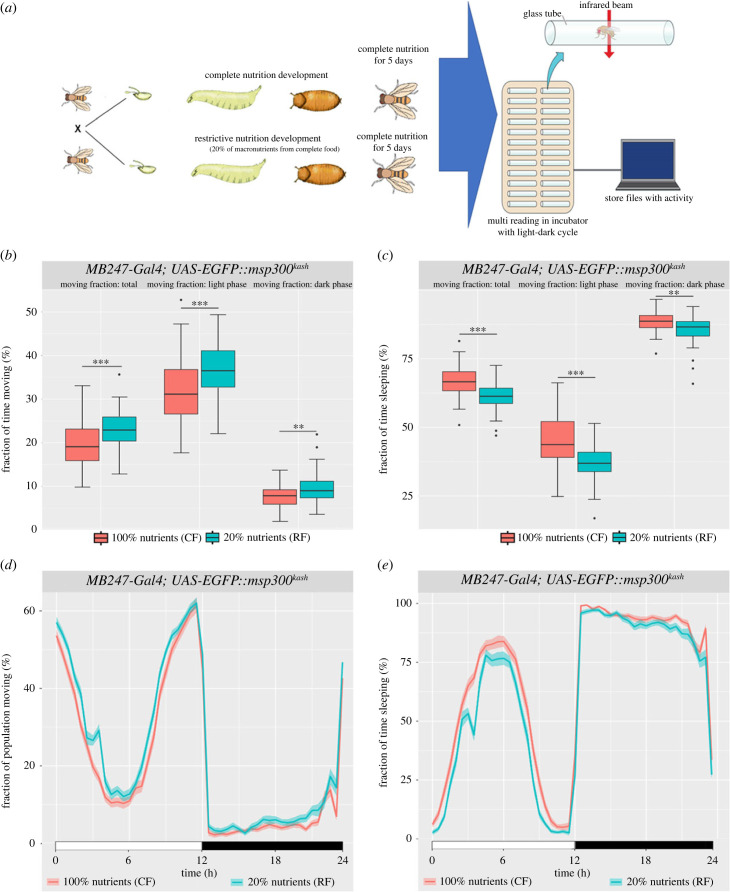
Restrictive nutrition during larvae development leads behaviour alterations in adults. (*a*) Experimental design showing the main genotype used in the study, together with the experimental model of nutrient restriction (restrictive food, RF) and the control condition (complete food, CF). The TriKinetics measuring system diagram was adapted from Harper [28]. (*b*) Box plots depicting the fraction of time spent moving during the total day, light phase and dark phase. The measures were obtained from 30 min windows, considering a fly as moving when it passed through the detector at least once per minute. (*c*) Box plots depicting fraction of time spent sleeping, with sleep defined as the absence of activity for at least 5 min, followed by continuous measurement of the duration of inactivity. (*d*) Fraction of the population with detected moving activity (measured in 30 min windows), plotted over 24 h with the thin colour representing the standard error. (*e*) Average fraction of time spent sleeping for each fly within a 30 min interval (measured from 5 min of inactivity) and plotted over 24 h. The thin colour represents the standard error. Sample size: *n* = 47 (RF), *n* = 54(CF). The genotype is indicated above the panels. Significance was calculated using the Mann–Whitney test: *p < 0.05, **p < 0.01, ***p < 0.0001.

First, we tested whether nutrient restriction during development generates measurable alterations in adult spontaneous locomotor activity and sleep behaviour. We compared the behavioural performance of adults generated from larvae reared in standard culture media (control food, CF) versus larvae reared in a culture media containing 20% of the total nutrients of the CF, referred to as restricted food (RF) (figure 1*a*). After hatching, both groups of adult flies were transferred to tubes with a filter paper soaked with liquid medium composed by 5% sucrose and 10% yeast extract.

The motor activity of 5-day-old virgin females was analysed individually with the DAM2-*Drosophila* activity monitor system (TriKinetics, Waltham, MA). We focus our analyses on the *MB247-Gal4; UAS-EGFP::msp300kash* genotype since it allowed us to perform the cell type-specific gene expression and chromatin opening analyses described later. In addition, we also used *oregon-rc* and *canton-s* fly genotype backgrounds to confirm our observations.

The locomotor activity analysis for the experimental genotype mentioned above shows that flies raised in RF (figure 1*b*, in cyan) are more active than flies raised in CF (figure 1*b*, in magenta). Remarkably, the higher activity showed by the RF group over the CF group was during the light phase of the day. Additionally, we tested locomotor activity in *oregon-rc* and *canton-s*, detecting similar changes in locomotor activity and sleep (electronic supplementary material, figure S1*a–d*).

As previously mentioned, spontaneous locomotor activity is higher in animals raised in RF than in CF. This effect on activity could also impact sleeping time. We found that adult flies grown in RF (cyan) sleep less than flies grown in CF (magenta, figure 1*c*), and this effect, as expected, is also stronger during the light phase of the day. Also, these changes are sustained over time, especially noticeable in all the light phase of the day (electronic supplementary material, figures S2 and S3). In addition, we measured main sleep parameters (electronic supplementary material, figure S4), in which only latency to first sleep event (bout) is altered in RF flies (electronic supplementary material, figure S4F).

These results suggest that early-life undernutrition has long-lasting impact on adult locomotor activity and sleep behaviour, specifically on the light phase of the day which appears to be the most sensitive period.

### 2.2. Transcriptional changes of adult mushroom body neurons in response to early-life nutritional restriction

The origin of adult behavioural changes could lie in gene expression effects that alter synaptic connectivity and axonal/dendritic extensions [11]. Changes in these features have a different impact on *Drosophila* behaviour, depending on which neuronal type or neuropils these changes occur. Since the MBs are directly related to associative learning and behaviours like feeding [19], olfactory memory [20,21], motor activity [18] and sleep [22,23], we set up to analyse the gene transcriptional profiles of adult MBs neurons in both dietary conditions.

**Figure 2.**
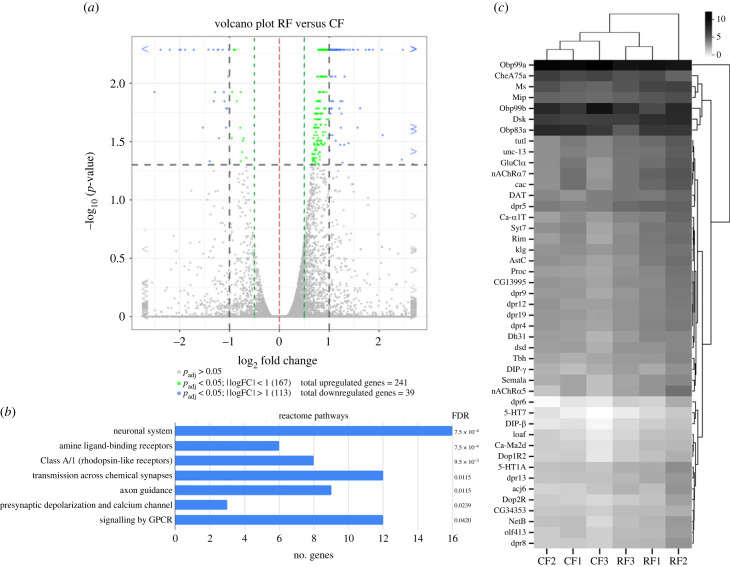
mRNA-seq for comparison of transcript levels in RF and CF from mushroom bodies’ nuclei. (*a*) Volcano plot depicting the significative genes, fold change and adjusted *p*-value (*p*_adj_) distributions. Significative genes were identified as *p*_adj_ < 0.05 and |log2(fold change)| > 1.5. This cut off gives 280 significant expressed genes (RF versus CF) with 239 upregulated genes and 41 downregulated genes. (*b*) Reactome pathways identified from upregulated genes. Seven pathways from 66 genes were identified, most of them related directly with neuronal functions, no reactome pathways were detected with the downregulated genes group. (*c*) Heatmap from genes related with neuronal functions (extracted from reactome pathways, excluding redundant genes, and supplemented with downregulated genes related with neuronal processes, giving 45 for the final list). The scale was constructed from FPKM values (fragment per kilobase of mapped reads), normalized with log2(FPKM + 1), then clustered.

To isolate Kenyon cell nuclei, first, we expressed a UAS transgene coding for a nuclear membrane-tagged EGFP protein (*UAS-EGFP::msp300kash*) in MBs neurons (specifically *α*/ß and *γ* neurons) using the *MB247-Gal4* driver [29]. Then we immunoprecipitated the nuclei from adult brain homogenate using an anti-EGFP antibody. After checking for EGFP mRNA expression enrichment compared with total nuclei from brain homogenate (electronic supplementary material, figure S5), total RNA was extracted to generate libraries for mRNA-seq and to perform differential expression analysis between RF and CF. We use 1.5X fold change (or log_2_(fold change) > [0.5]), e-value < 0.05 and FDR < 0.05 for significance as cutoff criteria. With these cutoff parameters, 280 differentially expressed genes were identified, with 241 of them (86%) being upregulated and the remaining 39 (14%) being downregulated (Volcano plot, figure 2*a*; see electronic supplementary material, file S1 for complete list of genes and sequencing statistics). Most genes that change their expression are related to neuronal processes in Reactome pathways (figure 2*b*; electronic supplementary material, file S2 for complete GO analysis). A group of 45 genes is directly related to the nervous system which are depicted in a heatmap (figure 2*c*). Taking a closer look at enrichment in Gene Ontology (GO) categories, we found upregulated genes in the category of ‘synapse organization’ (GO:0050808, 12 genes), ‘dopamine receptor signalling pathway’ (GO:0007212, 2 genes), ‘dopamine catabolic process’ (GO:0042420), ‘cation transport’ (GO:0005261, 5 genes) and ‘adult locomotory behaviour’ (GO:0008344). These gene clusters suggest that MB neurons could have increased excitability, neurotransmitter reception (dopamine signalling) and transmission to output neurons (mushroom body output neurons, MBONS). We found that dopamine receptors *dop1R2* and *dop2R*, which are highly expressed in MBs [25,27,30], are upregulated in response to our developmental nutritional restriction protocol. These data suggest that MBs could be relevant in the behavioural response to nutrition restriction through dopamine signalling.

Another interesting group of upregulated genes in neuroblasts subjected to nutrient restriction includes *alk* and *jeb,* which are necessary to keep on the insulin pathway despite the lack of extracellular Dilps [4]. These genes remain upregulated in adulthood, indicating that these neurons, and probably others in the brain, retain a transcriptional signature that makes them sustain metabolism in the face of poor nutrient availability.

In summary, we found groups of genes that increased their expression in RF, which according to their GO classification, could explain the alterations in behaviour. These expression changes in adults may result from early modifications in the configuration of the transcriptional machinery in neuroblast's chromatin in response to nutrition restriction.

### 2.3. Chromatin accessibility patterns of adult mushroom body neurons change in response to nutrition restriction

There is evidence of chromatin accessibility changes in neurons in response to different conditions [31], such as nutrition [32] and neuronal activity [33]. Thus, the epigenetic configuration and chromatin accessibility changes could serve as a molecular memory to sustain the variations in gene expression underlying the behavioural alterations observed in adulthood. ATAC-seq (assay of transposase accessible chromatin) [34] allows us to characterize chromatin opening globally and, therefore, to determine whether there are different chromatin configurations between MB neurons from animals raised under different nutrient availability.

**Figure 3.**
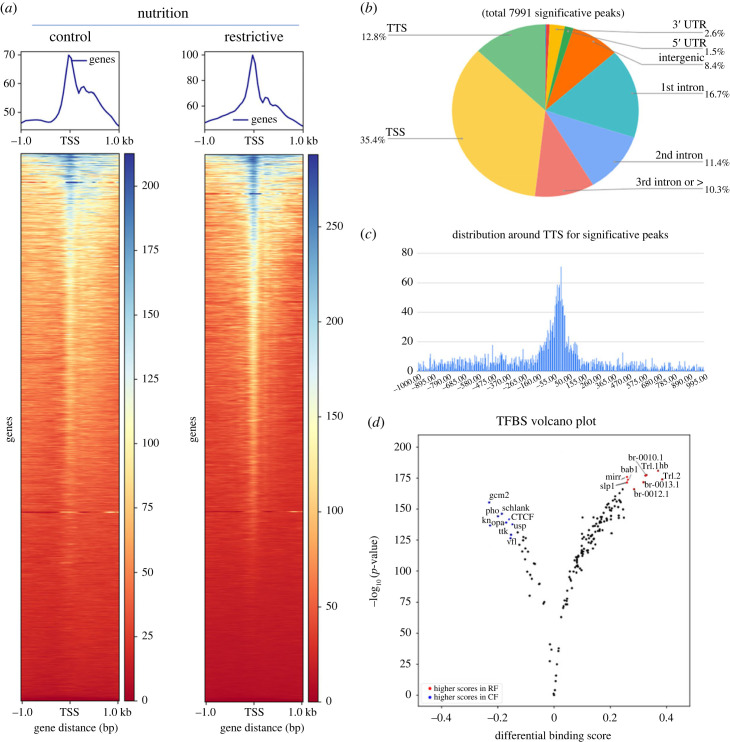
Atac-seq experiment from mushroom body neurons. Nuclei were isolated from *mb247 > Gal4; UAS-EGFP::msp300kash* whose samples were collected from conditions in complete nutrition (CF) and nutritional restriction (RF). Metagene profile around TSS and heatmap for the two conditions using alignment data, showing a clearly tendency of distribution around TSS. (*b*) Summary of significative peaks (open chromatin) identified with analysis, including exclusive peaks from every condition. (*c*) Distribution of significative peaks around TSS. (*d*) Volcano plot from analysis with TOBIAS showing the TFBS score for 160 binding sites for opened chromatin sites with significative footprinting. The TFBS with significative score are in blue for CF and red for RF.

We found clear patterns of open chromatin state on promoters and transcription start sites (TSS) on the genome in each diet (figure 3*a*). Open chromatin zones were identified as windows or regions between 10 and 100 bp and compared between the two nutritional conditions to identify peaks exclusive to RF. We identified 7991 exclusive peaks for RF distributed with high percentages within the region (−1000, +50) containing the promoter and transcriptional start site (TSS), and the first intron (figure 3*b*,*c*; see electronic supplementary material, S3 Data for full annotation), genomic zones usually associated with transcriptional regulation [35]. We annotated the open chromatin sites associating the regulatory features mentioned above to a particular gene [36] and, with this information, performed a GO analysis (electronic supplementary material, S4 Data). Also, we found an enrichment of KEEG pathways, such as Hippo signalling, metabolism, FoxO pathway and the circadian rhythm (table 1).

**Table 1.** KEEG pathways from new open chromatin sites in restrictive food condition (RF) annotation with HOMER. Complete GO analysis for new open chromatin sites in RF condition are in electronic supplementary material, file S4.

term	count	%	*p-*value
dme04391:Hippo signalling pathway - fly	31	0.7333806482138633	5.4622464410323125 × 10^−5^
dme01230:Biosynthesis of amino acids	30	0.7097232079488999	9.35248602176056 × 10^−4^
dme00600:Sphingolipid metabolism	15	0.35486160397444994	0.00827403246078938
dme01210:2-Oxocarboxylic acid metabolism	11	0.2602318429145966	0.010104961739882894
dme04320:Dorso-ventral axis formation	14	0.33120416370948663	0.01084080159891861
dme04068:FoxO signalling pathway	23	0.5441211260941565	0.012689000462610747
dme04711:Circadian rhythm - fly	7	0.16560208185474332	0.015337890499886328
dme04013:MAPK signalling pathway - fly	10	0.23657440264963334	0.02140948135221997
dme04310:Wnt signalling pathway	34	0.8043529690087533	0.024945785362360726
dme00561:Glycerolipid metabolism	20	0.4731488052992667	0.026101016157044365
dme04745:Phototransduction - fly	12	0.28388928317955997	0.0521269088114878
dme00564:Glycerophospholipid metabolism	23	0.5441211260941565	0.05692326893416723
dme04070:Phosphatidylinositol signalling system	19	0.44949136503430326	0.07639121970564666
dme04350:TGF-beta signalling pathway	15	0.35486160397444994	0.08460224800666333

Since most new chromatin open sites locate in transcriptional regulatory zones, we searched for transcription factor binding sites (TFBS) footprints and their changes between nutritional conditions. We performed a binding-site shift analysis with TOBIAS (Transcription factor Occupancy prediction By Investigation of ATAC-seq signal) [37], using a matrix of 160 TFBS extracted from JASPAR database [38]. We found significative changes in binding for 9 TFBS in RF (figure 3*d*, see red dots; full analysis is in electronic supplementary material, file S5). Two TFBS correspond to the Trithorax-like transcription variants (Trl.1 and Trl.2), whose binding sites directed the complexes to favouring open chromatin for transcription, possibly by overcoming the repressive effect of polycomb group complex [39,40]. Thus, these results suggest that the chromatin configuration settled in the larvae stage could favour transcription activation over repression in the MB neuronal precursors. These results are consistent with our mRNA-seq results, which showed more upregulated than downregulated genes. Moreover, in RF, we observed an enrichment of open chromatin sites which contain the Bab1 binding site (figure 3*d*), a TF required for olfactory neuron development. Interestingly, silencing *bab1* gene in MB during development generates smaller alpha/beta lobules in this neuropil [41], supporting the conjecture that chromatin configuration influences the transcription of genes related to neuronal identity, contributing to the behavioural changes observed in flies reared under nutrient restriction.

**Figure 4.**
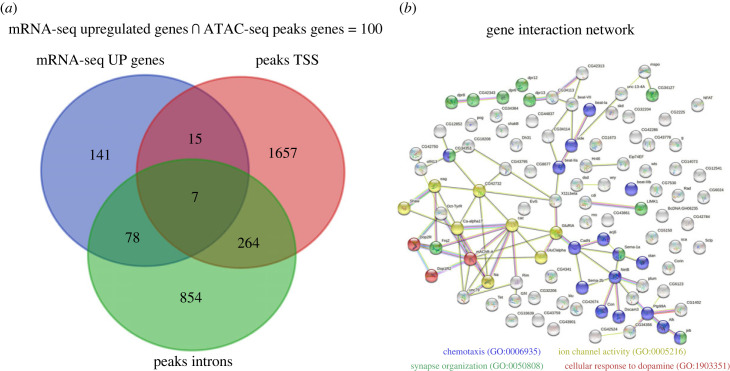
Cross data between ATAC-seq (open chromatin sites in RF) and RNA-seq (upregulated genes in RF). (*a*) Venn diagram between RNA seq Upregulated genes and peaks (open chromatin) in Transcription Start Site (TSS, −1000 bp, +100) and introns. (*b*) Gene interaction network generated with String. Every circle represents a gene with colours for different or common GO category: blue for chemotaxis (GO:0006935); green for synapse organization (GO:0050808); yellow for ion channel activity (GO:0005216); red for cellular response to dopamine (GO:1903351). Nodes filled with protein figure indicated there is a three-dimensional model. Lines between nodes indicate know interaction from known databases (cyan), know interactions experimentally determined (magenta), predicted interaction between genes by neighbourhood (green), predicted interaction by gene fusions (red), predicted interaction by co-occurrence (blue), text mining (yellow), co-expression (black) and protein homology (light blue).

We found 100 out of the 241 upregulated genes (41%) map to ATAC-seq open chromatin sites in TSS, introns, or both (figure 4*a*). GO analysis from this cross data show significant enrichment in chemotaxis, excitability (expressed as voltage-dependent channels) and dopaminergic transmission (figure 4*b*; table 2).

**Table 2.** GO for Biological process from cross data in figure 5. Complete analysis from these data are in electronic supplementary material, file S6.

no. term ID	description	observed gene count	background gene count	strength	FDR
GO:0007399	nervous system development	31	1050	0.62	9.82 × 10^−8^
GO:0030182	neuron differentiation	25	682	0.71	1.10 × 10^−7^
GO:0048666	neuron development	22	544	0.76	2.58 × 10^−7^
GO:0008038	neuron recognition	12	115	1.17	3.71 × 10^−7^
GO:0006935	chemotaxis	16	270	0.92	3.91 × 10^−7^
GO:0022008	neurogenesis	27	895	0.63	3.91 × 10^−7^
GO:0048699	generation of neurons	26	854	0.63	5.22 × 10^−7^
GO:0032501	multicellular organismal process	54	3449	0.34	5.33 × 10^−7^
GO:0048731	system development	36	1661	0.48	6.66 × 10^−7^
GO:0007411	axon guidance	15	251	0.92	8.18 × 10^−7^
GO:0061564	axon development	16	308	0.86	1.22 × 10^−6^
GO:0007154	cell communication	30	1258	0.53	2.58 × 10^−6^
GO:0023052	signalling	29	1190	0.54	3.00 × 10^−6^
GO:0098742	cell–cell adhesion via plasma-membrane	9	69	1.26	3.76 × 10^−6^
GO:0098609	cell–cell adhesion	10	99	1.15	4.48 × 10^−6^
GO:0048468	cell development	29	1263	0.51	9.02 × 10^−6^
GO:0007155	cell adhesion	11	165	0.97	3.57 × 10^−5^
GO:0050808	synapse organization	11	175	0.95	5.52 × 10^−5^
GO:0000904	cell morphogenesis involved in differentiation	16	433	0.72	6.39 × 10^−5^
GO:0007275	multicellular organism development	38	2270	0.37	8.27 × 10^−5^
GO:1903351	cellular response to dopamine	3	21	1.30	0.029
GO:0005216	ion channel activity	8	242	0.67	0.042

### 2.4. Pharmacological and genetic attenuation of dopamine signalling in adulthood reverse the behavioural phenotype induced by developmental nutrition restriction

**Figure 5.**
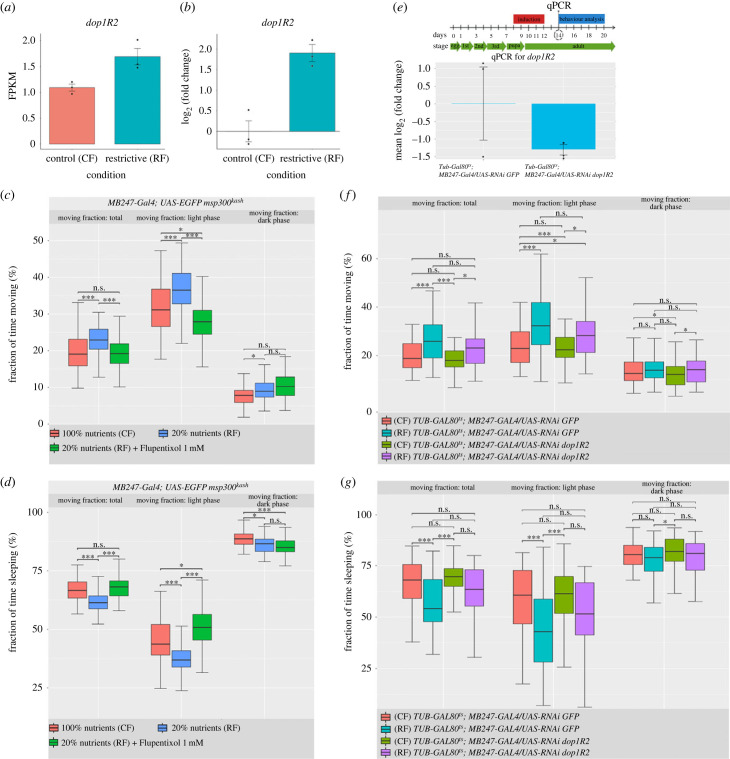
Attenuation of dopamine signalling diminishes the effect of RF in behaviour. (*a*) *dop1R2* FPKM values from mRNA-seq in mushroom bodies’ nuclei. For the mRNA-seq, the significance was measured by *t*-test *p*_adj_ < 0.05 between FPKM values and for qPCR the significance was estimated with *t*-test *p*-value < 0.05 for dCt method comparison (*b*) dop1R2 Fold change values (in log2) from mRNA measured with qPCR. (*c*) Locomotor activity measured as fraction of time moving using pharmacological treatment with flupentixol. (*d*) Sleep time measured as fraction of time sleeping using pharmacological treatment with flupentixol. (*e*) Experimental design for the induction of *dop1R2* RNAi indicating the induction time (29°C) and the time of RNA extraction to validate the effect of RNAi induction on *dop1R2* expression. dCt method was used to estimate significance with *p* < 0.05 for the comparison RNAi versus Control (*f*) Locomotor activity measured as fraction of time moving to compare the effect of *dop1R2* knockdown on the movement. (*g*) Sleep time measured as fraction of time sleeping to compare the effect of *dop1R2* knockdown on sleeping time. *a priori* significance was calculated with Kruskal–Wallis test, with *p*-value < 0.0001 for mb247 > Gal4; UAS-EGFP:: msp300kash. For pair comparison, Dunn's test was applied with: *p < 0.05, **p < 0.01, ***p < 0.0001. *n* = 51 (CF), *n* = 47 (RF); *n* = 56 (RF + Flupentixol treatment) for mb247 > Gal4; UASEGFP:: msp300kash (panels *c* and *d*); *n* = 68 (CF *Tub-Gal80ts; MB247-Gal4/UAS-GFP RNAi*); *n* = 67 (RF *Tub-Gal80ts; MB247-Gal4/UAS-GFP RNAi*; *n* = 53 (CF *Tub-Gal80ts; MB247-Gal4/UAS-dop1R2 RNAi*); *n* = 59 (RF *Tub-Gal80ts; MB247-Gal4/UAS-dop1R2 RNAi*) (panels *f,g*).

Since chromatin accessibility and mRNA-seq reveal dopaminergic signalling as a relevant component of the response to RF, and that KCs have the highest expression of dopamine receptors in the adult *Drosophila* brain [25,27,30], we reasoned that these neurons signal more frequently and intensely due to increased dopaminergic signalling in RF, affecting spontaneous activity and sleep behaviour (figure 1*b*,*c*). Also, we confirmed the increment in Dopamine receptor *dop1R2* which is up regulated in mRNA-seq (Figure 5*a*) with qPCR using the same isolation nuclei method (Figure 5*b*), giving a log2(fold change) over 1.5 (or>3.6X as mean, with 1.14 s.d.).

To test whether decreasing dopamine signalling could reverse the behavioural effects produced by RF, we use Flupentixol, which is a dopamine antagonist tested previously in insects [42–44]. We treated adult flies supplementing with a low concentration (1 mM) of Flupentixol [44] the same liquid medium, in filter paper and for the same time, as treatment used in figure 1*b*,*c*. Afterwards we measured spontaneous activity and sleep behaviour. Remarkably, Flupentixol treatment was able to restore the locomotor activity (figure 5*c*; electronic supplementary material, figure S6) and the sleep behaviour (figure 5*d*; electronic supplementary material, figure S7) of adult flies raised under RF to the levels of CF flies. Of note, the effect of Flupentixol was more evident in *MB247 > Gal4; UAS-EGFP::msp300^kash^* genetic background than in the *oregon-rc* background (electronic supplementary material, figure S8). The restoration of the behavioural phenotypes was more potent during the light phase of the day than in the dark phase, supporting the premise of increased excitability to the stimulus occurring in the light phase.

Considering an alternative and more specific approach to prove the point related to dopamine signalling, we tested if knockdown of *dop1R2* in mushroom body neurons could reverses the altered behaviour at some level. This dopamine receptor, whose mRNA expression increased (figure 5*a,b*) in RF conditions, also with new chromatin open regions in the regulatory area (intron)*,* is a good candidate to mediate potential changes in the dopamine signalling in MBs, considering in addition that it is almost exclusively expressed in MBs neurons [25]. RNAi expression was induced exclusively in adult MB neurons, using the *Tub-Gal80ts; MB247-Gal4* genotype*,* which makes it possible to activate the Gal4/UAS system exclusively at 29°C. The diagram of this experiment is presented in figure 5*e*, it is worth mentioning that the control treatment was also subjected to temperature induction, in this case the control RNAi targets GFP. As observed in figure 5*f,g*, *dop1R2* RNAi expression in MB neurons of animals raised in RF produced minor changes in adult behaviour, both in locomotion and sleep, suggesting that dopamine signalling through this receptor, specifically in MB neurons is highly relevant for maintaining these long-term behavioural changes, which are based at the transcriptional and epigenetic level.

Altogether, the results demonstrate how low-nutrient availability during development, but not fasting, generates adult flies with alterations in locomotion and sleep, even if those individuals were fed with high nutrients during adulthood. Examining transcription profiles and chromatin states in MBs neurons, we were able to associate these behavioural alterations with an increment of dopamine signalling within this circuitry, which is responsible for locomotion, memory and sleep.

## 3. Discussion

In humans, malnutrition during prenatal development is a frequent condition in poor and developing countries [45], generating as long-term psychiatric problems that involves cognitive function decline [7], higher risk of schizophrenia [12] and premature brain ageing [46]. Since sleep alterations are frequently found in schizophrenia and in other mental disorders, we propose that *Drosophila melanogaster* could be an excellent model to evaluate the mechanisms by which developmental malnutrition affects adult behaviour leading to mental illness. Here, we show that a restrictive nutrition during the larval stages of *Drosophila* development results in increased spontaneous activity and in a reduction of sleep time in adults.

### 3.1. High locomotion and diminished sleep time are adaptations to increase foraging

*Drosophila* larvae [47–49] and adults [50] increase foraging activity as a natural survival response. These behavioural changes for increasing locomotion, foraging and exploring activity are widely conserved among insects [51] and birds [52].

In our results, the increase in activity is more evident during daylight hours (light phase), when these animals typically feed (figure 1*b*; electronic supplementary material, figure S1A and B). During this period, effector neurons and temperature receptors are more sensitive due to the circadian cycle [53]. The circadian cycle plays a relevant role in nutrition, mainly affecting behaviour [54] and metabolism [55]. A free-running experiment would be relevant to understand if adult flies from malnutrition larvae exhibit the same alterations and examine the impact on circadian rhythm.

Our results show that, in accordance with the locomotor alterations, adult *Drosophila* flies from larvae raised in nutrition restriction sleep less than flies raised in normal nutrition conditions (figure 1*c*; electronic supplementary material, S1C and S1D). Similarly, adult flies maintained under starvation conditions generate shorter sleep times during the day [56]. However, in our experiments, the adults are maintained under regular nutritional conditions, suggesting that reduced sleep and increased spontaneous activity are long-lasting compensatory effects.

These compensatory effects could be associated with food search and feed, which are necessary to gain biomass but not grow after the eclosion. Also, there is evidence that larvae with nutrition low in calories and macronutrients, but with normal nutrition later during adulthood, increase their biomass exhibiting macronutrient preference, without increasing in external body size [57]. This increase occurs in the fat body and other internal organs as the reproductive system, especially in female flies [57], meaning these alterations in behaviour could be coupled with the entire homeostasis of the adult animal.

### 3.2. Early-life nutrition imprints epigenetic and transcriptional changes in genes associated with excitability and connectivity in Kenyon cells

The open chromatin analysis combined with mRNA-seq led us to propose a group of genes whose transcription is regulated directly from the open/close chromatin state. Interestingly, most of those genes have functions related with processes that would explain the behavioural phenotype (figure 4; table 2). On the other hand, we found 141 upregulated genes without an obvious change in open chromatin state in their *loci*, which could be explained by changes in mRNA dynamics, such as rates of degradation/stability [58]. These phenomena cannot be detected with our mRNA-seq approach, since it sequences the pool of mRNA at the moment of its extraction, omitting information about its kinetics.

Interestingly, we found a greater number of new open chromatin sites in TSS than genes that upregulated their expression. Histone bivalence may explain this result. Equal presence of H3K4me3 (related to transcriptional initiation) and H3K27me3 (related to transcriptional repression) on these promoters and TSS [59,60], in which the ATAC-seq detects an opening but still transcription does not progress. The bivalent promoters have been documented in development for fast response to morphogens and some stress adaptation [61,62]. However, further analysis is required to demonstrate what exactly happens in the chromatin areas that open without a detectable change in expression. Alternatively, transcriptional pausing may explain the imbalance between expression and open chromatin state. In this case, chromatin is kept open in TSS zone, and RNA Pol II is arrested short after transcription initiation, remaining inactive until new signals are sensed, such as morphogens or heat stress [63]. Based on this, the early-life nutrition restriction may set up chromatin configurations and transcriptional pausing, enabling fast transcriptional responses when nutrient availability changes or to face subsequent environmental challenges. This mechanism could explain why psychiatric diseases derived from *in utero* malnutrition are triggered in adulthood [12] and not during childhood. More work will be necessary to test this proposal.

We showed that early-life undernutrition impacts chromatin state and gene expression in the adult MBs neurons, affecting excitability and dopamine signalling (figure 4). Dopamine is a well-studied neurotransmitter, and its signalling pathway is widely conserved among animals. In *Drosophila* dopamine biosynthesis pathway involves tyrosine and L-Dopa as intermediaries [64], and its genome comprises four G-couple protein receptors well-characterized: Dop1R1, Dop1R2, Dop2R and DopEcR [26], all expressed in MB neurons [65].

The modulation of motor behaviour by dopamine has been well documented in *Drosophila*, as well as its role in MB neurons [43,66]. Since Dop1R1 and Dop1R2 are expressed in the KCs [25,27,43], changes in their transcription could result in a natural adaptation to increase motor activity and foraging during daylight. This last hypothesis is supported by results showing that MB dopamine receptors are necessary to maintain wakefulness exclusively during daylight [67], agreeing with our behavioural results (figure 1*b*,*c*; electronic supplementary material, figure S1). However, dopamine signalling is complex in the MB; Dop1R1 can reduce motor activity [66], while Dop1R2 plays an activating role, specifically in the optomotor response [68]. Together these data led us to propose that Dop1R2 is the most relevant receptor in the change of behaviour detected in this work, supported by the upregulation in its transcripts (figure 5*a,b*), the open chromatin configuration in this gene (figure 4*b*) and also considering the fact that its expression is specific in KCs [25].

### 3.3. Attenuation of dopamine signalling diminishes the effect of RF in behaviour

Based on the above results, which suggest an important role for dopamine signalling, we pharmacologically inhibit this pathway, supplementing the diet of adults with Flupentixol, a dopamine receptor antagonist. This treatment moderates the effects of early-life undernutrition, decreasing the effects on spontaneous activity and sleep behaviour (figure 5*c,d*; electronic supplementary material, figures S6 and S7). These results led us to propose that pharmacological treatment of adults can ameliorate the behavioural alterations generated in response to undernutrition during development. Therefore, dopaminergic disbalance in this context may be considered in treating psychiatric disorders which could have some basis *in utero* malnutrition. The role of dopamine in maintaining the altered behaviour under developmental nutrition restriction as previously mentioned could be further narrow down to DopR2. The knockdown of *dop1R2* in adult MBs neurons, confirmed that reducing the expression of this receptor in these specific neurons is sufficient to generate the partial reversion in the behaviour phenotype, revealing the key role of the epigenetic and transcriptional regulation of this receptor in MBs neurons in mediating the changes derived from poor nutrition in development.

In summary, we analysed the behaviour of adult *Drosophila* grown under developmental conditions of general nutrient shortage, leading to alterations such as higher locomotor activity and decreased sleep, especially in the daylight phase. These effects may derive from changes in chromatin accessibility that are associated with the expression of genes related to neuronal excitability, dopamine signalling and metabolic adaptation. Considering dopamine signalling, our observations show that the drug Flupentixol suppress the behavioural effects that were correlated with the epigenetic configuration set during the neuroblast stage. Furthermore, it was verified that Dop1R2 dopamine receptor, specifically expressed in MBs neurons, has an important role in the altered behaviour derived from the low-nutrient diet at initial developmental stage. Our findings give insights to understand similar processes in humans and other animals, especially those linked to psychiatric diseases, which would have a basis in developmental malnutrition or epigenetic imprinting, so that by understanding the base of those changes, new treatments could be considered.

## 4. Methods

### 4.1. Behavioural analysis and food

Female-virgin flies were analysed on Dam2-Trickinetics system on tubes with 5% sucrose on one side and measured the movement for 5 days, using the last 2.5 days for the main analysis. Data extraction and analyses were performed with *Rethomics* software for R [69], and plots were generated with *ggetho2* software for R. Larvae were developed in two conditions: one with complete culture media as CF or cultured in restrictive nutrition food (RF) with 1/5 of nutrients in CF. CF was composed by beer yeast 10% w/v, sucrose 5% w/v, 1.3% w/v agar, 6% v/v propionic acid. RF was composed by beer yeast 2% w/v, sucrose 1% w/v, 1.3% w/v agar, 6% v/v propionic acid. Activity monitors in vials were maintained in a 12 h:12 h light-dark (LD) cycle. In the first 5 days of life before the behavioural analysis, adult flies were fed in filter paper over agar, with 5% w/v sucrose and yeast extract 10% w/v, and optionally with 1.0–1.5 mM of Flupentixol.

### 4.2. Fly stocks

For MBs nuclei tag expression, we use *UAS-EGFP::Msp-300^KASH^*^,^ Ma and cols. [70] under the Gal4 driver on *MB247 > Gal4* (Bloomington stock 50742) for specific MBs expression. Other behaviour analyses were done with flies *oregon-rc* (Bloomington stock 5) and *canton-s* (Bloomington stock 64349). For Knockdown experiments, a *dop1R2* RNAi from the TRiP collection (Bloomington stock 26018), and a control RNAi against GFP (Bloomington stock 35786) were used. These stocks were crossed with *+; Tub-Gal80^ts^; MB247-Gal4* to perform a temperature inducible expression.

### 4.3. mRNA and chromatin extraction from mushroom body neurons

For ATAC-seq and transcriptomic analysis, 200 female flies expressing EGFP::Msp-300^kash^ per replicate were frozen on liquid nitrogen to detach the heads after a brief vortex. The heads were homogenized at 4°C on isolation nuclei extraction buffer [70] (10 mM HEPES-KOH, pH 7.5; 2.5 mM MgCl_2_; 10 mM KCl), in an eppendorff tube with polypropylene tip, then kept for 15 min on ice, and then 20 pulls with a polypropylene tip. The homogenized was filtered on a cell strainer (40 µm) and then the nuclei were isolated with a centrifuge at 2000 rpm for 5 min. The nuclei were resuspended on 1 ml of IP buffer, then incubated for 1 h with 5 µg of EGFP antibody (abcam ab290) and 50 µl of Dynabeads-Protein A (Thermo scientific). The nuclei were isolated with a magnet to remove supernatant and 500 µl of Trizol was added. Samples were vortexed and 0.1 µl of chloroform was added. The mixture was vortexed and centrifuged at 14 000 rpm (4°C, 15 min). The supernatant was recovered and RNA was precipitated with 0.8 volume of isopropanol and pelleted at 14 000 rpm (4°C, 15 min), washed once with 70% ethanol and pelleted again. Ethanol was removed, and the pellet was resuspended on 20 µl of molecular biology water. RNA concentration was measured for quantity and quality. Libraries for mRNA-seq were generated with CATS mRNA-seq kit from Diagenode, according to instructions of the manufacturer. Libraries were sequenced on Illumina Hiseq 2500. The same isolation procedure was performed for qPCR analysis.

For ATAC-seq libraries, we follow the same steps as mRNA-seq libraries until before Trizol addition. Instead of Trizol, isolated nuclei were resuspended on Tn5-mix from Nextera Illumina kit, then following instruction of manufacturer. Samples were sequenced on Illumina Hiseq-2500 with paired-end, 75 bp.

### 4.4. Sequencing analysis

mRNA-seq paired-end reads were trimmed with Trimmomatic and analysed with Cuffdiff [71] and annotated with HOMER [36], with comparison parameters 1.5X, at *p*-value < 0.05, e-value < 0.05. ATAC-seq raw data was filtered with Trimmomatic [72] and paired-end reads were aligned with Bowtie2 [73] (*Drosophila* Release 6 plus ISO1 MT-dm6, using the follow parameters: –dovetail; –maxins 1000; –very-sensitive filtering duplicates, mitochondrial mapping and alignment quality. Once the Bam mapping files were filtered and checked for insert sites, we select just one replicate per condition. Then perform peak calling for open chromatin with MACS2 using the bed12 file (parameters: –nomodel; extension size 200; shift size –100; No broad regions; sophisticated signal processing approach to finding subpeak summits in each enriched regions). After peak calling, the significant peaks were annotated with HOMER [36]. To detect differential open chromatin sites between CF and RF conditions, the software Bedtools (subtract method) was used [74]. To identify TFBS, TOBIAS was used [37] with the scripts for correction of ATAC-seq bias and subsequent analysis of all binding sites for 160 TF (ATACorrect, ScoreBigwig and BINDetect).

## Data Availability

Illumina data from mRNA-seq and ATAC-seq are available in NLM BioSample collection under the accession PRJNA891475. The data are provided in electronic supplementary material [75].
